# TMT-Based Proteomic Explores the Influence of DHEA on the Osteogenic Differentiation of hBMSCs

**DOI:** 10.3389/fcell.2021.726549

**Published:** 2021-08-17

**Authors:** Xiaonan Liang, Mingwei He, Bo Zhu, Yongjia Zhu, Xixi He, Dachang Liu, Qingjun Wei

**Affiliations:** ^1^Department of Orthopedics Trauma and Hand Surgery, The First Affiliated Hospital of Guangxi Medical University, Nanning, China; ^2^Guangxi Collaborative Innovation Center for Biomedicine, Guangxi Medical University, Nanning, China; ^3^Guangxi Engineering Center in Biomedical Materials for Tissue and Organ Regeneration, The First Affiliated Hospital of Guangxi Medical University, Nanning, China; ^4^Nanning Second People’s Hospital, The Third Affiliated Hospital of Guangxi Medical University, Nanning, China

**Keywords:** hBMSCs, DHEA, osteogenic differentiation, TMT, bioinformatic analysis

## Abstract

Dehydroepiandrosterone (DHEA) has been revealed to implicate in facilitating osteoblast differentiation of human bone marrow mesenchymal stem cells (hBMSCs) and inhibiting osteoporosis (OP). However, the underlying molecular mechanism remains largely unknown. Here, we induced osteogenic differentiation of hBMSCs derived from elders using an osteogenic induction medium with or without DHEA. The results showed that osteogenic induction medium (OIM) with DHEA could significantly promote the proliferation and osteogenic differentiation of hBMSCs than OIM alone. By using a Tandem Mass Tag (TMT) labeling and liquid chromatography-tandem mass spectrometry (LC-MS/MS) technology, we screened out 604 differentially expressed proteins (DEPs) with at least one unique peptide were identified [524: OIM vs. complete medium (CM), and 547: OIM+DHEA vs. CM], among these proteins, 467 DEPs were shared in these two different comparative groups. Bioinformatic analysis revealed these DEPs are mainly enriched in metabolic pathways. Interestingly, the expression levels of the DEPs in the metabolic pathways showed a more noticeable change in the OIM+DHEA vs. CM group than OIM vs. CM group. Moreover, the protein-protein interaction (PPI) network analysis revealed that three potential proteins, ATP5B, MT-CYB, and MT-ATP6, involved in energy metabolism, might play a key role in osteogenic differentiation induced by OIM+DHEA. These findings offer a valuable clue for us to better understand the underlying mechanisms involved in osteoblast differentiation of hBMSCs caused by DHEA and assist in applying DHEA in hBMSCs-based therapy for osteogenic regeneration.

## Introduction

Osteoporosis (OP) is a systemic disorder that is characterized by bone fragility, accompanied by a sharp decrease in bone strength (Ginaldi et al., 2005; Franco-Trepat et al., 2019). With increasing age, these bone losses are raised, in turn resulted in a higher risk of fragility fractures (Lynn et al., 2005; Chen et al., 2019). Thus, the morbidity of OP increases with the aging of the population, severely affecting the quality of life and physical health of the elderly (Chen et al., 2019). According to statistics, osteoporosis-induced disabling injuries have become the third most serious disease affecting the life and health of the elderly following hypertension, cardiovascular, and cerebrovascular diseases (An et al., 2016; Tan and Dai, 2019). However, although the approved anti-osteoporosis drugs currently have some therapeutic effect on the treatment of osteoporosis, the risk of osteonecrosis of the jaw and atypical femoral fractures was increased after long-term intake, thereby leading to their limited utility in clinical (Voss et al., 2018). Hence, it is urgent to develop new therapeutic strategies for OP.

Mesenchymal stem cells derived from human bone marrow (hBMSCs) are multipotent stromal cells that can differentiate into osteoblasts, chondrocytes, adipocytes, and many other cell types (Pittenger et al., 1999). It is currently believed that proliferation and differentiation of hBMSCs, especially the osteogenic differentiation, have close correlations with the onset and recovery of OP, providing new ideas for the treatment and study of the disease (Wang et al., 2007, 2009). Furthermore, in recent years, the regenerative medical potential of human mesenchymal stem cells (hMSCs), specifically bone marrow-derived MSCs, have attracted increasing attention, and these cells were used frequently for augmenting bone tissue repair and regeneration in bone tissue engineering, and were considered as a promising tool for bone regeneration in OP (Ciapetti et al., 2006; Annamalai et al., 2019; Liu et al., 2019). Hence, enhancing the proliferation and osteogenesis differentiation of BMSCs and understanding the underlying mechanisms may significantly improve the therapeutic efficacy of OP.

Dehydroepiandrosterone (DHEA) and its sulfate ester, the prominent adrenal steroids in human circulation, are used as a nutritional supplement in the United States and can be obtained without a prescription. DHEA is touted for its putative anti-aging peculiarities and potential advantage in metabolic and cardiovascular health (Allolio and Arlt, 2002; Formoso et al., 2006). Recent studies reported that DHEA could accelerate the growth of osteoblast and morphometry of bone tissue, and oppose osteoporosis by inhibiting certain activities of endogenous glucocorticoids (GC) in rodents (Malik et al., 2010). DHEA has been reported to promote the proliferation of osteoblast and inhibit apoptosis of osteoblast and maturation of osteoclast. In addition, DHEA significantly boosts the production of osteoblast by modulating the expression of genes associated with osteoblast in MSC (Qiu et al., 2015). Our previous study evaluated the effect of DHEA on hBMSCs and showed that the higher ALP activity was recorded at 10 nM of DHEA in the medium (Liang et al., 2016). As the literature grew, DHEA will become widely used as a promising agent to treat osteoporosis by affecting the osteogenic differentiation of hBMSCs. However, systemic approaches for specifying the influence of DHEA on osteogenic differentiation of hBMSCs have not yet to be investigated, and the molecular mechanism of osteogenic differentiation remains unclear. Proteins, as the actual functional molecules involved in various biological effects in the human body, should be translated from messenger RNA and then undergo a variety of modifications before they can be used as the available proteins. Therefore, the expression of biological molecules at the genomic level alone cannot wholly reflect the truth.

Recently, proteomics methodology has been employed for the investigations of a series of mesenchymal stem cells (MSCs), including the differential expression profile analysis of membrane proteins in MSCs during differentiation of osteoblast (Foster et al., 2005), the analysis of the secretome of MSCs differentiated from embryonic stem cells (Sze et al., 2007), the proteomic analysis of rat MSCs subcultures (Celebi and Elçin, 2009), and proteomic profiling of transforming growth factor-β acting on MSCs (Wang et al., 2004; Kurpinski et al., 2009). Nevertheless, data on the proteome profile of bone tissue-derived MSCs was still limited, and further studies are needed to provide information for better application of these populations of MSCs. Therefore, to gain further understanding of the underlying molecular mechanism of the osteogenic differentiation of hBMSCs, we applied a Tandem Mass Tag (TMT) peptide labeling combined with liquid chromatography-tandem mass spectrometry (LC-MS/MS) approach to quantitatively evaluate the protein expression profile of the osteogenesis model *in vitro*. The TMT labeling coupled with LC-MS/MS is a gel-free quantitative proteomics technology that enables isobaric labeling of proteins and was used for the quantification and identification of biological macromolecules, such as peptides and proteins, which is one of the most sensitive techniques currently used for quantitative analysis of proteomes (Zhang and Elias, 2017).

Herein, we explored the promote effects of DHEA on the osteogenic differentiation of hBMSCs, and analyzed differentially expressed proteins (DEPs) of hBMSCs cultured in three different mediums under the two-dimensional system using TMT peptide labeling combined with LC-MS/MS technology. We particularly focused on the differential expression of proteins during osteoblast differentiation of hBMSCs induced by osteogenic induction medium (OIM) and OIM with DHEA and conducted bioinformatics analysis. The DEPs identified in this study may serve essential roles in the progression of osteogenic differentiation. Our findings provide an important insight into the crucial proteins and the underlying intracellular mechanisms of osteogenic differentiation, further promoting their application of DHEA in OP therapy.

## Materials and Methods

### Extracting and Culturing of hBMSCs

All studies involving human stem cells were approved by the Ethics Committee of The First Affiliated Hospital of Guangxi Medical University. HBMSCs were isolated from the iliac crest of donors who received a total hip replacement for osteoporosis in the Department of Orthopedics Trauma and Hand Surgery, The First Affiliated Hospital of Guangxi Medical University. Patients gave informed written consent before they participated in this study. The criteria for exclusion have been explained in our previous reports (Liang et al., 2016; Zhou and Glowacki, 2017). Briefly, the mononuclear cells were extracted from bone marrow that had been treated with heparin by using Ficoll-Hypaque density gradient centrifugation with a density of 1.077 g/L. Subsequently, the cells were seeded in a six-well plate and cultured with α-minimal essential medium (α-MEM, Gibco-BRL, United States) containing 10% fetal bovine serum (FBS, Gibco-BRL, United States) and 100 units/mL penicillin and 100 μg/mL streptomycin (Solarbio, China), which is called as complete medium (CM), and then incubated in an incubator (Thermo Fisher Scientific, United States) at 37°C with 5% CO_2_ humidified atmosphere. After 7 days of culture, the hBMSCs were harvested and re-cultured in 10 cm of plastic dishes at the density of 4 × 10^3^ cells/cm^2^, and the cultured medium was replaced with a fresh medium every 3 days. The trypsin-EDTA solution containing 0.02% EDTA and 0.25% trypsin (Solarbio, China) was applied to detach the cells, and a subculture was conducted at 1:3 split when the cells reached 100% confluence. The cells from passage three were used for further experimentation.

### Cytotoxicity Assay

To determine the toxic effect of DHEA on hBMSCs, the cytotoxicity was detected by an MTT (Gibco-BRL, United States) assay following the manufacturer’s instruction. In brief, the hBMSCs with a density of 1,000 cells/well were cultured in a 96-well plate. After 3 days of treatment with DHEA with a variety of concentrations (0–100 μM of DHEA where 0 μM was employed as a control), and the plates were incubated at 37°C in the dark for 4 h after the addition of 20 μL of MTT reagents (5 mg/ml) into each well. After removing MTT reagents, 200 μL of dimethyl sulfoxide (DMSO, Sigma-Aldrich, United States) was added to the cells for crystal solubilization. After that, the spectrometric absorbance at the wavelength of 570 nm was obtained by Multiskan^TM^ GO microplate spectrophotometer (Thermo Fisher Scientific Inc., United States). Every experiment was performed in triplicate. An optimal concentration of 10 nM was chosen for further investigation according to the results of MTT analysis.

### Treatment and Grouping of hBMSCs

The hBMSCs were divided into three groups as follows: CM group: hBMSCs were cultured with complete medium; osteogenic induction medium (OIM) group: hBMSCs were induced with OIM (Shen et al., 2019), which composed of α-MEM, 10% FBS, β-glycerophosphate (10 mM) (Sigma-Aldrich, United States), dexamethasone (10 mM), and ascorbic acid-2 phosphate (50 μg/mL); OIM+DHEA group: hBMSCs were cultured in OIM containing 10 nM of DHEA.

### Alkaline Phosphatase Activity Test

The cultured cells were lysed with RIPA lysis buffer (Beyotime, China) after wash twice with Phosphate Buffer Saline (PBS). Subsequently, the Alkaline Phosphatase Assay Kit (Beyotime, China) was used to assess the cellular alkaline phosphatase (ALP) activity quantitatively in the light of the manufacturer’s protocol. After that, the cellular ALP activity was measured under the wavelength of 405 nm by using a microplate reader (Thermo Fisher Scientific, United States).

### Alizarin Red Staining for Calcium Deposit

After 7 and 14 days of culture, the process of fixation with 4% paraformaldehyde was carried out after the cultured cells were washed twice with PBS. Subsequently, the cells underwent evaluation of calcium levels using alizarin red staining according to the standard protocols. Light microscopic images were observed and captured using an Olympus BX53 microscope (Olympus, Japan).

### Real-Time Quantitative Polymerase Chain Reaction Analysis

Total RNA was purified using an RNA isolation kit (Tiangen Biotech, China) according to the manufacturer’s specifications. The cDNA was synthesized from the RNA by using a reverse transcription kit (Takara, Japan). The real-time quantitative polymerase chain reaction (qRT-PCR) was performed with Fast Start Universal SYBR Green Master Mix (Roche, Germany) in the procedure with 40 cycles of 95°C for 15 s, 60°C for 30 s, following by 72°C for 30 s by using ABI Step One Real-Time PCR System machine. Every reaction was repeated three times. The relative expression levels of target genes were measured by employing 2^–ΔΔCT^ method. The sequences of gene primer employed for the amplification of the target gene were presented in Table 1.

**TABLE 1:** Primer used in the qRT-PCR.

**Gene name**	**Forward primer**	**Reverse primer**
**ALPL**	GCAAGAAAGGGGACCCAAGA	CAGAATGTTCCACGGAGGCT
**COL1A1**	GCTTCACCTACAGCGTCACT	AAGCCGAATTCCTGGTCTGG
**RUNX2**	TGTCATGGCGGGTAACGATG	CCCTAAATCACTGAGGCGGT
**BMP2**	TCCATGTGGACGCTCTTTCA	AGCAGCAACGCTAGAAGACA
**GAPDH**	CTATAAATTGAGCCCGCAGC	GACCAAATCCGTTGACTCCG

*ALPL, Alkaline phosphatase; COL1A1, Collagen type I alpha 1 chain; RUNX2, RUNX family transcription factor 2; BMP2, Bone morphogenetic protein 2; GAPDH, Glyceraldehyde-3-phosphate dehydrogenase.*

### Proteomic Analysis by TMT

Human bone marrow mesenchymal stem cells were cultured in a 55 cm^2^ plastic culture dish at a density of 10,000 cells/cm^2^ with a culture medium as formerly indicated. The cells were lysed with 2 mL of cell lysis buffer after detachment with trypsin when 14 days of growth. Subsequently, the protein concentrations were determined using a Bradford Quant Kit (Amresco) according to the manufacturer’s protocol. And then, 100 g of each protein sample was treated with 3.3 L of trypsin (1 g/L) at 37°C for 24 h, and following by labeled with the TMT. After desalting of the TMT-labeled peptides by C18 reversed-phase column, the lyophilized products were further separated by using the Dionex capillary/nano-HPLC system, and then transported to the Q-Exactive Plus spectrometer for further analysis. The mass spectrum was obtained by the software Proteome Discoverer 2.1 in a data-dependent manner. The analytical cycle consisted of a single full-scan mass spectrum (350--2000 Da), followed by 15 data-dependent MS/MS scans. Under this scan, to generate reporter ions and fragment ions, TMT-labeled peptides were fragmented. The ratio of reporter ions reflected the relative abundance of the peptides or proteins in the sample. Fragment ions were applied to identify the labeled peptide sequences and their corresponding proteins. The data acquired in the TMT experiments were analyzed with Mascot 2.5 using the UniProt-Homo Sapiens database^1^.

### Bioinformatics Analysis

Compared with the Control group, the proteins with a threshold of fold change (FC) more or less than 1.2 and *p*-value less than 0.05 were defined as differentially expressed proteins (DEPs). The functional classifications, pathway enrichment, and Gene Ontology (GO) term analysis on the DEPs were conducted after the identification of proteins using the bioinformatics method. By using the Gene Ontology analysis database^2^, GO enrichment analyses classified proteins based on biological process, cell component, and molecular function. The pathway analysis of the DEPs was carried out applying the Kyoto Encyclopedia of Genes and Genomes (KEGG) online database^3^. KEGG pathways and GO terms with corrected *p* < 0.05 were considered significant enrichment.

### Western Blot Analysis

The western blot analysis was performed as previously described (Ma et al., 2020). Briefly, hBMSCs were seeded at a density of 10,000 cells/cm^2^ in six-well plates and allowed to grow for 14 days in different mediums as the former indicated. After that, cells in the diverse group were harvested to detect the expression of ATP5B, MT-CYB, and MT-ATP6. For extraction of proteins, RIPA buffer (Beyotime, Shanghai, China) was used to lyse the cells. The concentrations of proteins were determined using A BCA protein assay kit (Beyotime, China). Furthermore, 60 micrograms of each protein lysate were resolved using 10% SDS polyacrylamide gel and transferred to polyvinylidene difluoride membranes (Millipore, Bedford, MA, United States). The membranes were then incubated at 4°C overnight with an appropriate concentration of primary antibodies as follows: ATP5B (1:500, Proteintech, Catalog No. 17247-1-AP), MT-CYB (1:500, Proteintech, Catalog No. 55090-1-AP), MT-ATP6 (1:500, Proteintech, Catalog No. 55313-1-AP), and β-actin (1:1000, Proteintech, Catalog No. 66009-1-Ig). After washing the membrane three times for 10 min with 1× TBST, the membranes were incubated with a secondary antibody on a rocking platform for 30 min at room temperature. Finally, the membranes were exposed to Odyssey infrared imaging system (LI-COR Biosciences, Lincoln, NE, United States) for the appropriate period to acquire the images. Quantitative analysis of the band intensity was analyzed by Image J.

### Statistical Analysis

The statistical analysis of data was carried out using GraphPad Software (GraphPad Prism 5.0). All values are presented as the mean ± standard deviation (SD). The data were evaluated using one-way ANOVA and the Tukey’s *t*-test using SPSS 16.0 (IBM, United States). *P*-value < 0.05 was considered statistically significant.

## Results

### The Optimal Concentration of DHEA Without Cytotoxicity and Contributes to the Proliferation of hBMSCs

**FIGURE 1:**
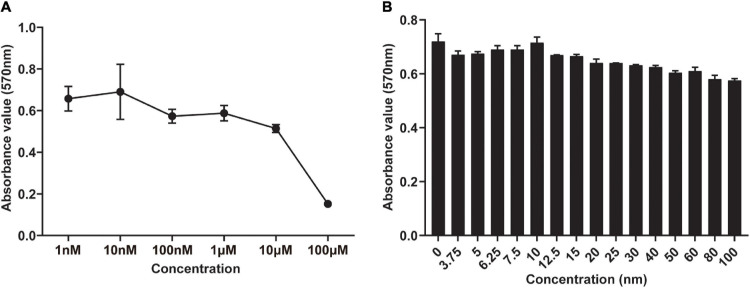
Screening of the optimal concentration of DHEA in the viability of hBMSCs by MTT assay. **(A)** The viability of hBMSCs was measured after 3 days of co-culture with DHEA at various concentrations (0–100 μM). **(B)** The viability of hBMSCs was measured after 3 days of co-culture with DHEA at multiple concentrations (0–100 nM).

To evaluate the cytotoxicity of DHEA toward hBMSCs and its effect on the proliferation of BMSCs, we conducted an MTT assay. In the traditional complete medium, no apparent cytotoxicity was observed in the hBMSCs treated with DHEA at a range of 1–10 nM for 3 days, but a gradually reduce cell viability was observed at higher concentrations (100 nM–100 μM) (Figure 1A). It is worth noting that cell viability was significantly increased in the hBMSCs treated with DHEA at a range of 6.25–10 nM, a concentration of 10 mM arrived a peak increase in viability, where thereafter, there was a gradual decline in vitality at higher concentrations (Figure 1B). These results indicated that 10 nM is the lowest and most proper functional concentration of DHEA on hBMSCs. Therefore, the concentration of 10 nM for DHEA was selected for subsequent investigations.

### DHEA Promotes Osteoblastic Differentiation of hBMSCs *in vitro*

**FIGURE 2:**
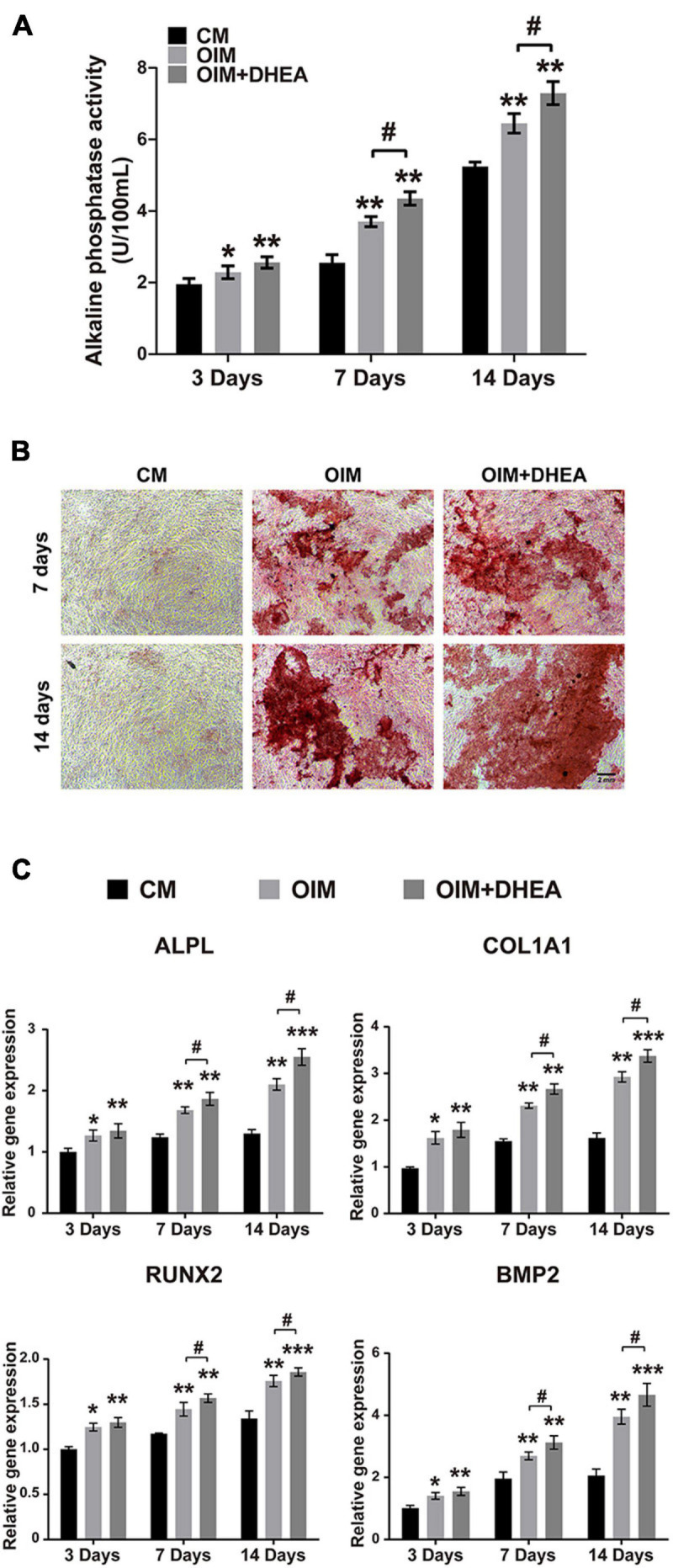
DHEA promotes the osteogenic differentiation of BMSCs *in vitro*. **(A)** ALP activity was analyzed after 3, 7, and 14 days of hBMSCs culturing with CM, OIM, and OIM supplemented with 10 nM of DHEA. Error bars: mean ± SD. *N* = 3, *, # indicates *p* < 0.05, **indicates *p* < 0.01. **(B)** Alizarin red staining of osteogenic hBMSCs treated with or without 10 nM of DHEA for 7 and 14 days (Scale bar = 2 mm). **(C)** The mRNA expression of Alpl, Col1a1, Runx2, and Bmp2 was detected in the CM, OIM, and OIM+DHEA groups at the indicated time by qRT-PCR analysis. Error bars: mean ± SD. *N* = 3, *, # indicates *p* < 0.05, **indicates *p* < 0.01, ***indicates *p* < 0.001.

To explore the functions of DHEA on osteoblastic differentiation, we performed a quantitative alkaline phosphatase (ALP) assay and Alizarin red staining when hBMSCs were cultured in the osteogenic induction medium (OIM) with or without DHEA for 3, 7, and 14 days, respectively. As shown in Figure 2A, our results showed no significant difference in ALP activity observed in the OIM group and OIM+DHEA group at 3 days. However, the ALP activity in the OIM+DHEA group was significantly higher than that in the OIM group at 7 days and 14 days. Meanwhile, alizarin red staining yielded comparable results, where the formation of calcification deposits was markedly increased in the OIM+DHEA group compared with the CM and OIM group at 7 days and 14 days (Figure 2B). In addition, the expression levels of osteoblast-specific genes, Bone morphogenetic protein-2 (Bmp2), Runt-related transcription factor 2 (Runx2), Collagen type I alpha 1 (Col1a1), and Alkaline phosphatase (Alp) were detected in hBMSCs treated with 10 μM of DHEA for 3, 7, and 14 days by RT-PCR analysis. As shown in Figure 2C, compared to the hBMSCs cultured in CM, the mRNAs expression level of Col1a1, Runx2, Bmp2, and Alp was markedly increased in the OIM group and OIM+DHEA group. What’s more, the expression of osteogenic-specific genes was dramatically elevated after 7 and 14 days of DHEA treatment compared with the hBMSCs cultured in OIM without DHEA. The above results suggested that the addition of DHEA is more effective than conventional inducer in inducing osteogenic differentiation of hBMSCs.

### Identify the DEPs in OIM and OIM+DHEA Groups Through TMT Proteomic Analysis

**FIGURE 3:**
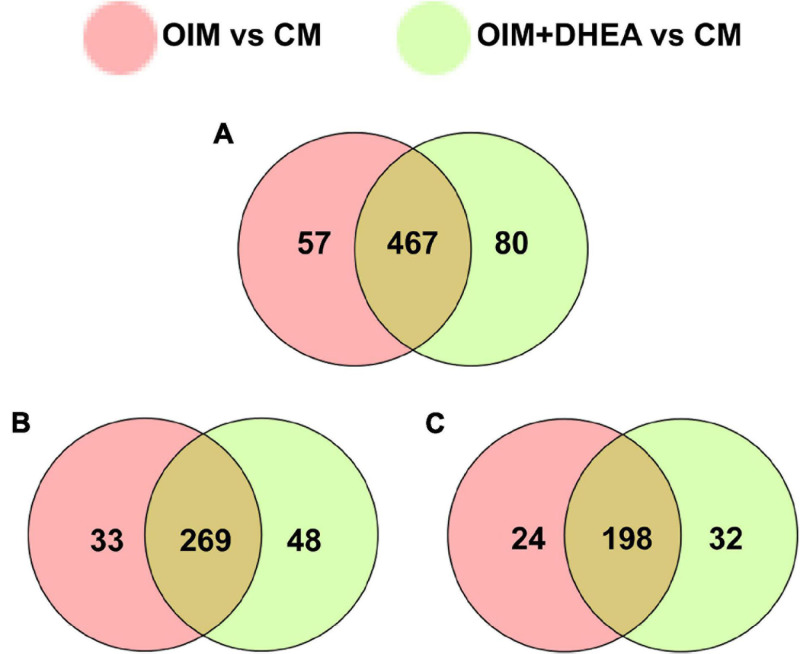
Analysis of differentially expressed proteins (DEPs) between two different comparative groups. **(A)** Venn diagram of DEPs comparing between OIM vs. CM and OIM+DHEA vs. CM. **(B)** Venn diagram of up-regulated proteins comparing between OIM vs. CM and OIM+DHEA vs. CM. **(C)** Venn diagram of down-regulated proteins comparing between OIM vs. CM and OIM+DHEA vs. CM.

To explore the patterns of proteome during the osteogenic differentiation of hBMSCs induced by DHEA, comparative proteome analysis was performed in hBMSCs cultured with CM, OIM, and OIM+DHEA by using TMT. Under the condition of FC > 1.2 or FC<−1.2 and *P* value < 0.05, a total of 45614 spectra were obtained in the comparison between OIM and CM (OIM vs. CM), and 50590 spectra were obtained in the comparison between OIM+DHEA and CM (OIM+DHEA vs. CM). By searching UniProt-Homo Sapiens database, we identified 604 proteins containing at least one unique peptide from these spectra (Table 2). Among these proteins, the number of differentially expressed proteins (DEPs) between OIM and CM groups was 524, containing 222 down- and 302 up-regulated proteins. However, there were 547 DEPs, including 230 down- and 317 up-regulated proteins in OIM+DHEA vs. CM. And 467 DEPs were shared in these two different comparative groups, including 269 up- and 198 down-regulated proteins (Figure 3).

**TABLE 2:** Quantitative results of protein.

**Comparisons**	**Spectra**	**Peptides**	**Proteins**		
			**Up-**	**Down-**	**All**
**OIM vs. CM**	45,614	3,348	302	222	524
**OIM+DHEA vs. CM**	50,590	3,633	317	230	547

*FC > 1.2 or FC < −1.2 and *P* value < 0.05.*

### Gene Ontology Enrichment Analysis

**FIGURE 4:**
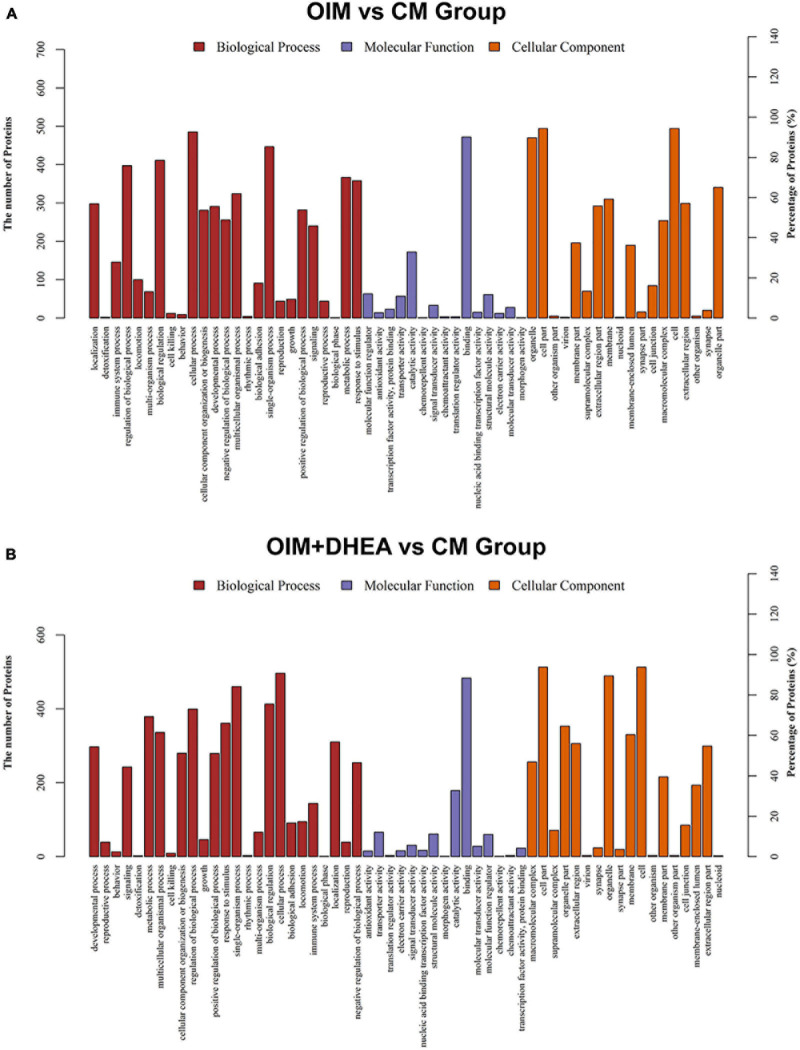
Gene ontology (GO) classification of differentially expressed proteins (DEGs). GO analysis of DEGs in OIM+DHEA vs. CM group **(A)** and OIM+DHEA vs. CM group **(B)**. Cut-off value: *p*-value < 0.05, and count >2.

Base on the Gene Ontology (GO) database, we investigated the enrichment of DEPs during osteogenic differentiation induced by two different induction mediums. In the OIM vs. CM, DEPs are mainly enriched in 25 GO terms, 15 GO terms, and 18 GO terms of the biological process (BP), molecular function (MF), and cellular component (CC), respectively. In the BP category, cellular process, single-organism process, and biological regulation were the top three terms. The first three terms of the MF category were catalytic activity, binding, and molecular function regulator. Cell, cell part, and organelle dominated the CC category (Figure 4A). These results indicated that OIM promotes the differentiation of hBMSCs into the osteoblasts might through promoting the activities of cell and organelle, and regulating the cellular process and binding-related molecular functions.

Interestingly, in the OIM+DHEA vs. CM, DEPs were enriched in similar GO terms in the BP, MF, and CC categories, separately. The most enriched three terms in the BP category were cellular process, regulation of biological process, and single-organism process. Transporter activity, catalytic activity, and binding were highly clustered terms in the MF category. The most significant three terms in the CC category were also the same as OIM+DHEA vs. CM, which was cell, cell part, and organelle (Figure 4B). These results suggested that DEPs in OIM vs. CM and OIM+DHEA vs. CM had a similar biological function in BP, MF, and CC categories.

### Kyoto Encyclopedia of Genes and Genomes Pathway Enrichment Analysis for the DEPs

**FIGURE 5:**
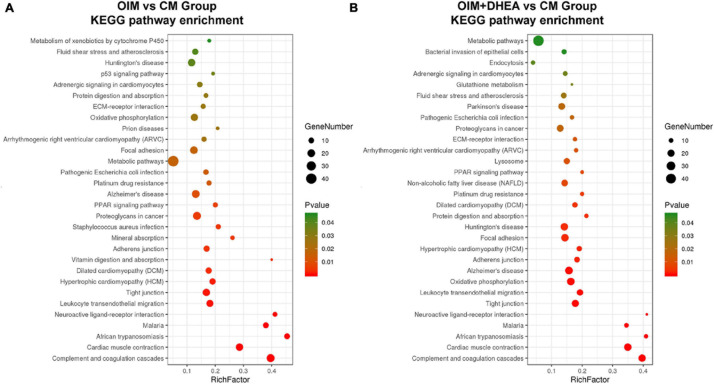
The enriched KEGG pathways of differentially expressed proteins (DEGs). The top 30 enriched KEGG pathways of the DEPs in OIM vs. CM **(A)** and OIM+DHEA vs. CM **(B)**. Cut-off value: *p*-value < 0.05.

To map the DEPs to the reference pathways and uncover their unique biological significance, the pathway enrichment analysis was carried out by employing the Kyoto Encyclopedia of Genes and Genomes (KEGG) database. As shown in Figure 5, among the top 30 enriched pathways (ranked by count), 24 pathways were identical, including Metabolic pathways, PPAR signaling pathway, Alzheimer’s disease, Oxidative phosphorylation, Huntington’s disease, and Focal adhesion in OIM vs. CM group and OIM+DHEA vs. CM group. Notably, among the 24 shared pathways, more proteins were enriched in the Metabolic pathways. A total of 30 proteins in the OIM vs. CM group and 37 proteins in the OIM+DHEA vs. CM group were involved in the metabolic pathways (Table 3). Of note, the top 20 DEPs in both OIM vs. CM group and OIM+DHEA vs. CM group all were enriched in the metabolic pathways, indicating that the metabolic pathways were more specific for osteogenic differentiation than other pathways. These results suggested that DHEA and OIM had similar molecular mechanisms, especially metabolic pathways, during osteogenic differentiation.

**TABLE 3:** Proteins enriched in metabolic pathways.

**Group**	**Enriched genes**
**OIM vs. CM**	CHIA, RFK, ATP6, COX1, COX2, ATP8, CYTB, UGP2, PFAS, AMY1A, ATP6V0C, UQCR11, ATP5L, LDHA, ALDOA, ARG1, ALPL, COX4I1, MAOA, ATP6V0C, ATP5D, NNMT, PRPS1, EXT1, Q59E93, Q6ZMU0, HGD, CYP2S1, HEL-S-271, HEL-S-41, HSD11B1
**OIM+DHEA vs. CM**	CHIA, RFK, GUK1, ATP6, COX1, COX2, ATP8, CYTB, UGP2, PFAS, AMY1A, ATP6V0C, PLPP1, UQCR11, ATP5L, LDHA, ALDOA, ARG1, ALPL, COX6C, COX4I1, MAOA, ATP6V0C, ATP5D, NNMT, UQCRFS1, XDH, PRPS1, ATP6AP1, EXT1, Q59E93, P4HA3, HGD, CYP2S1, HEL-S-271 (ATP5B), HEL-S-41, HSD11B1

### Analysis of Differentially Expressed Proteins in Metabolic Pathways

To further investigate the critical proteins that participated in metabolic pathways, the intersection of these proteins enriched in the metabolic pathways in each group was analyzed, and 29 shared proteins were harvested. As shown in Table 4, the changing trend of metabolic pathways protein was consistent between the two groups, and the change of most differentially expressed proteins in OIM + DHEA vs. CM group was more significant. Moreover, it’s worth noting that alkaline phosphatase (ALPL, a specific protein of biomineralization) was implicated in the metabolic pathways and was located on both the OIM+DHEA vs. CM and OIM vs. CM. Notably, compared with group OIM vs. CM, the expression of the ALPL was higher in group OIM+DHEA vs. CM, which is consistent with the result of qRT-PCR analysis (Figure 2C). Collectively, we concluded that DHEA could activate the metabolic pathways more effectively than OIM, thereby accelerating osteogenic differentiation.

**TABLE 4:** Common DEPs associated with metabolic pathways in OIM vs. CM and OIM+DHEA vs. CM groups.

**Accession**	**Gene name**	**Description**	**Coverage%**	**OIM vs CM**	**OIM+DHEA**
			**(unique peptide)**		**vs CM**
**Up-regulated**					
P05186	ALPL	Alkaline phosphatase, tissue-nonspecific isozyme OS = Homo sapiens GN = ALPL PE = 1 SV = 4	59.16	2.69	3.00
B7ZMD7	AMY1A	Alpha-amylase OS = Homo sapiens GN = AMY1A PE = 2 SV = 1	5.87	6.24	5.23
P05089	ARG1	Arginase-1 OS = Homo sapiens GN = ARG1 PE = 1 SV = 2	5.59	5.17	5.20
P30049	ATP5D	ATP synthase subunit delta, mitochondrial OS = Homo sapiens GN = ATP5D PE = 1 SV = 2	13.69	2.50	2.67
O75964	ATP5L	ATP synthase subunit g, mitochondrial OS = Homo sapiens GN = ATP5L PE = 1 SV = 3	47.57	2.52	2.69
A0A059QB80	ATP6 (MT-ATP6)	ATP synthase subunit a OS = Homo sapiens GN = ATP6 PE = 4 SV = 1	4.42	2.63	3.00
P27449	ATP6V0C	V-type proton ATPase 16 kDa proteolipid subunit OS = Homo sapiens GN = ATP6V0C PE = 1 SV = 1	11.61	2.32	2.36
A0A059QPX7	ATP8	ATP synthase protein 8 OS = Homo sapiens GN = ATP8 PE = 3 SV = 1	29.41	3.01	2.94
A0A024R0D9	CHIA	Chitinase, acidic, isoform CRA_a OS = Homo sapiens GN = CHIA PE = 3 SV = 1	3.99	4.24	3.36
A0A059QJB6	COX1	Cytochrome c oxidase subunit 1 OS = Homo sapiens GN = COX1 PE = 3 SV = 1	6.04	2.85	2.96
A0A059RTG7	COX2	Cytochrome c oxidase subunit 2 (Fragment) OS = Homo sapiens GN = COX2 PE = 3 SV = 1	31.53	2.91	2.45
P13073	COX4I1	Cytochrome c oxidase subunit 4 isoform 1, mitochondrial OS = Homo sapiens GN = COX4I1 PE = 1 SV = 1	51.48	2.34	2.58
Q96SQ9	CYP2S1	Cytochrome P450 2S1 OS = Homo sapiens GN = CYP2S1 PE = 1 SV = 2	1.79	2.44	2.82
A0A059RS35	CYTB (MT-CYB)	Cytochrome b OS = Homo sapiens GN = CYTB PE = 3 SV = 1	4.21	2.60	2.66
Q16394	EXT1	Exostosin-1 OS = Homo sapiens GN = EXT1 PE = 1 SV = 2	4.69	3.77	2.50
V9HW31	HEL-S-271 (MT-ATP5B)	ATP synthase subunit beta OS = Homo sapiens GN = HEL-S-271 PE = 1 SV = 1	74.10	3.46	3.79
V9HWF8	HEL-S-41	Epididymis secretory protein Li 41 OS = Homo sapiens GN = HEL-S-41 PE = 2 SV = 1	14.72	11.32	11.75
Q93099	HGD	Homogentisate 1,2-dioxygenase OS = Homo sapiens GN = HGD PE = 1 SV = 2	9.44	4.28	4.02
X5D2L1	HSD11B1	Hydroxysteroid 11-beta dehydrogenase 1 isoform A (Fragment) OS = Homo sapiens GN = HSD11B1 PE = 2 SV = 1	19.18	2.29	2.61
P21397	MAOA	Amine oxidase [flavin-containing] A OS = Homo sapiens GN = MAOA PE = 1 SV = 1	48.39	3.41	3.17
P40261	NNMT	Nicotinamide N-methyltransferase OS = Homo sapiens GN = NNMT PE = 1 SV = 1	45.83	2.37	2.68
A8K8N7	PFAS	Phosphoribosylformylglycinamidine synthase (FGAR amidotransferase), isoform CRA_b OS = Homo sapiens GN = PFAS PE = 2 SV = 1	4.71	2.59	2.44
Q59E93	Q59E93	Aminopeptidase (Fragment) OS = Homo sapiens PE = 2 SV = 1	49.85	2.57	2.67
A0A024R275	RFK	Riboflavin kinase, isoform CRA_a OS = Homo sapiens GN = RFK PE = 4 SV = 1	6.79	2.27	2.39
A0A087WYS1	UGP2	UTP–glucose-1-phosphate uridylyltransferase OS = Homo sapiens GN = UGP2 PE = 1 SV = 1	50.79	2.60	2.80
O14957	UQCR11	Cytochrome b-c1 complex subunit 10 OS = Homo sapiens GN = UQCR11 PE = 3 SV = 1	21.43	3.19	3.92
**Down-regulated**					
P04075	ALDOA	Fructose-bisphosphate aldolase A OS = Homo sapiens GN = ALDOA PE = 1 SV = 2	71.98	0.38	0.38
P00338	LDHA	L-lactate dehydrogenase A chain OS = Homo sapiens GN = LDHA PE = 1 SV = 2	57.83	0.52	0.50
P60891	PRPS1	Ribose-phosphate pyrophosphokinase 1 OS = Homo sapiens GN = PRPS1 PE = 1 SV = 2	43.08	0.52	0.51

### Analysis of Protein-Protein Interaction Network for These Proteins Involved in Metabolic Pathways

**FIGURE 6:**
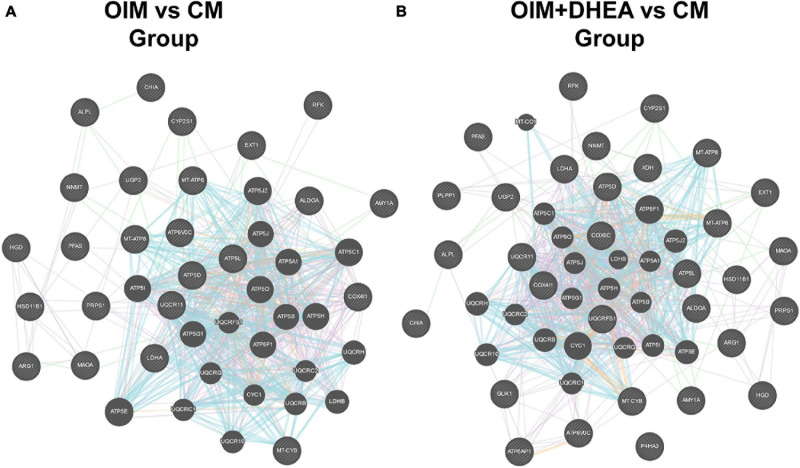
Protein-protein interaction network for the proteins in the metabolic pathways of OIM vs. CM group **(A)** and OIM+DHEA vs. CM group **(B)**.

**FIGURE 7:**
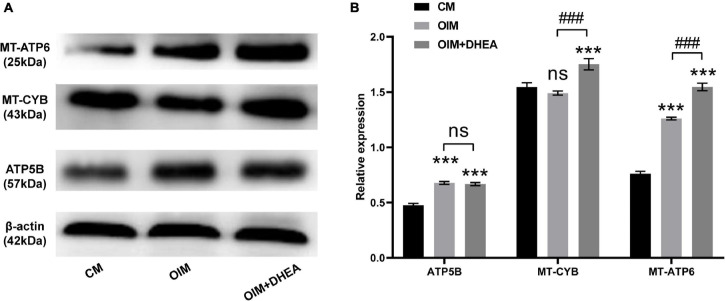
Protein expressions of ATP5B, MT-CYB, and MT-ATP6 were increased in the OIM group and OIM+DHEA group. **(A)** The expression of ATP5B, MT-CYB, and MT-ATP6 protein were assessed by western blot analysis, β-actin as a loading control. **(B)** The quantification of expression levels of ATP5B, MT-CYB, and MT-ATP6 proteins, the data were presented as the mean ± SD for each group (***p < 0.001; ###*p* < 0.001).

To further research the differences and similarities of the interaction of proteins involved in metabolic pathways in OIM vs. CM and OIM+DHEA vs. CM groups, the protein-protein interaction (PPI) network was constructed. As shown in Figure 6, we found that three proteins, ATP5B, MT-CYB, and MT-ATP6, are highly connected protein nodes, interacting with many more partners than average. Therefore, three proteins, ATP synthase subunit beta (ATP5B), Cytochrome b (MT-CYB), and ATP synthase subunit an (MT-ATP6), were considered to be key proteins in the whole network. Besides, combined protein expression profile, expression of ATP5B, MT-CYB, and MT-ATP6 was increased in group OIM+DHEA vs. CM compared with OIM vs. CM. In addition, to confirm the expression of ATP5B, MT-CYB, and MT-ATP6, we performed a western blot analysis. Indeed, the expression of ATP5B, MT-CYB, and MT-ATP6 proteins was markedly increased in the OIM group and OIM+DHEA group compared with that in the CM group. In particular, their expression in the OIM+DHEA group was significantly higher than that in the OIM group (Figure 7). These results indicated that three proteins, ATP5B, MT-CYB, and MT-ATP6, may play a key role in osteogenic differentiation induced by OIM+DHEA.

## Discussion

As a metabolic bone disease, the main feature of OP is loss of bone mineral density and high risk of fractures, in which osteogenesis of osteoblasts and bone resorption of osteoclasts is vital for the occurrence and development of OP (Wu et al., 2018). Therefore, it is critical to explore osteoblast differentiation of hBMSCs to provide novel and highly effective treatment options for OP. We found that DHEA could significantly improve osteoblast differentiation of hBMSCs *in vitro*. Although the research on osteogenic differentiation of hBMSCs induced by DHEA has provided significant insights into the occurrence, development, and therapy of OP, there are still outstanding insufficient in the mechanism research of osteogenesis. Thus, the identification and characterization of proteins that are expressed differently during the osteogenic differentiation of hBMSCs induced by DHEA might contribute to an in-depth understanding of the complexity of the osteogenic commitment and their therapeutic effect for OP.

In this study, we confirmed that DHEA addition could more significantly promote the proliferation and osteogenic differentiation of hBMSCs from elders than OIM alone, which agrees with the former research that DHEA stimulation promotes osteoblast differentiation (Malik et al., 2010; Qiu et al., 2015; Liang et al., 2016). DHEA has been reported to potentiate cell proliferation, differentiation, and osteocalcin production (Scheven and Milne, 1998). In addition, we also used a powerful proteomics tool, TMT, to investigate the underlying molecular mechanism of osteogenesis induced by DHEA. The TMT-labeled quantitative proteomics is an efficient and systematic method that improves the reliability of the data analysis to accurately quantifying relative protein levels. As untargeted research, TMT-labeled quantitative proteomics is increasingly being used to study the pathogenesis of a disease or cellular biological processes because of its ability to conduct systematic and unbiased analysis of various proteins in samples, as well as its advantages of high sensitivity and good reproducibility (Thompson et al., 2003; Moulder et al., 2018). Untargeted studies can provide a broader perspective on the molecular phenotypes of pathophysiological processes and have the prospect of studying a more comprehensive range of signal pathways (Ismail et al., 2019). Especially proteomics, untargeted studies of the proteome can provide some clues into the understanding of biological processes. For instance, Zhou et al. (2019) found multiple protein factors involved in the pathogenesis of osteoporosis through investigating protein alterations in the bone marrow microenvironment of osteoporotic patients undergoing spine fusion using TMT-based proteomics. Zhang et al. (2021) also applied TMT-based quantitative proteomic analysis to unveil the impact of BMSCs on hair follicle regeneration and confirm key hair regeneration-related protein targets in this therapeutic context.

To the best of our knowledge, this is the first time to apply this method to assess the underlying molecular mechanism in the osteogenic differentiation of BMSCs induced by DHEA *in vitro*. In our research, the more differentially expressed proteins of hBMSCs in the CM, OIM, and OIM+DHEA groups were obtained through TMT peptide labeling, combined with LC-MS/MS, as a set of 604 DEPs were identified from OIM vs. CM group (524 DEPs) and OIM+DHEA vs. CM group (547 DEPs). These proteins could play an important role in the osteoblast differentiation of hBMSCs induced by DHEA and may be closely related to osteogenesis.

Further bioinformatics analysis demonstrated that these differential proteins from OIM vs. CM group and OIM+DHEA vs. CM group were mainly enriched into metabolic pathways. Metabolic pathways have been reported to regulate the expression of genes via epigenetic modifications, and most of the cofactors, substrates, or allosteric regulators involved in epigenetic changes are produced by way of bioenergetic pathways (Motyl et al., 2017). Thus, control of metabolic pathways has a profound impact in controlling adenosine triphosphate (ATP) generation involved in energy metabolism and controlling the expression of genes, and these effects will affect the lineage determination of stem cells. Energy metabolism as one of the metabolic pathways has been confirmed to participate in osteoblast differentiation and osteogenesis (Smith and Eliseev, 2021). Increasing evidence revealed that substantial amounts of energy were necessary for the synthetic phase of osteoblast differentiation as osteoblasts required huge energy-producing capability (Dirckx et al., 2019). Together with our findings, these results support the fact that metabolic pathways, especially energy metabolism, play a vital role in the DHEA osteoinduction of hBMSCs.

Interestingly, we observed increased expression of proteins (ATP5B, MT-CYB, and MT-ATP6) related to oxidative phosphorylation (OXPHOS) in the OIM+DHEA vs. CM group compared with the OIM vs. CM group. Cumulating evidence has revealed that OXPHOS is a crucial supplementary pathway for ATP synthesis in energy metabolism (Wilson, 2017), and increasing evidence has recently announced that human MSCs (hMSCs) primarily generate ATP through OXPHOS-dependent metabolism during osteogenic differentiation (Guntur et al., 2014; Meleshina et al., 2016). Thus, we speculated that DHEA promotes osteogenic differentiation of hBMSCs may through regulating energy metabolism of metabolic pathways by up-regulating three OXPHOS-related proteins (ATP5B, MT-CYB, and MT-ATP6). ATP is mainly synthesized by mitochondrial ATP synthetase. As one of the principal subunits of ATP synthase, ATP5B could promote cellular ATP levels and play a key role in the differentiation and proliferation of stem cells (Xiaoyun et al., 2017). MT-ATP6 protein that is a component of a large enzyme was known as ATP synthase, is encoded by the mitochondrial genome and responsible for catalyzing the final step of oxidative phosphorylation (Andrews et al., 1999; Demir et al., 2014). MT-CYB protein, as the only mitochondrial genome-encoded subunit of respiratory complex III, is encoded by the MT-CYB gene and exerts important functions in the electron transport system (Demir et al., 2014). Although the precise role of each of these three proteins alone in osteogenic differentiation induced by DHEA currently not know, these observations point to the increased expression of ATP5B, MT-CYB, and MT-ATP6 observed in osteocytes differentiation of hBMSCs caused by DHEA may represent a regulated mechanism of mitochondrial function to up-regulate ATP synthesis by OXPHOS. Further investigations are needed to address the precise tasks of DEPs these possibilities in osteogenic differentiation of hBMSCs and determine whether ATP5B, MT-CYB, and MT-ATP6 cause changes in energy metabolism, which impact the differentiation of hBMSCs into osteocytes induced by DHEA.

In short, our findings suggested that DHEA possesses osteoinduction properties and could foster the proliferation and osteoblast differentiation of hBMSCs in OIM. The proteomics and bioinformatics analysis revealed that DHEA-induced osteogenic differentiation of hBMSCs mechanically is mainly through energy metabolism as one of the metabolic pathways. Three key proteins, ATP5B, MT-CYB, and MT-ATP6, may act as the essential regulators for promoting the osteogenic differentiation of hBMSCs, and their specific functions need to be further studied. Our results offered novel information about the underlying molecular mechanisms involved in osteogenic differentiation of hBMSCs induced by DHEA, and DHEA is likely to be an ideal reagent for the prevention and treatment of OP.

## Data Availability Statement

The datasets presented in this study can be found in online repositories. The names of the repository/repositories and accession number(s) can be found below: MassIVE database, accession no: MSV000087888.

## Ethics Statement

The studies involving human participants were reviewed and approved by the Ethics Committee of The First Affiliated Hospital of Guangxi Medical University. The patients/participants provided their written informed consent to participate in this study.

## Author Contributions

XL, MH, and BZ contributed to the design and conception of the research, analysis of the data, interpretation of the results, and the writing of the manuscript. YZ, XH, and DL performed some experiments, provided reagents, and critical comments on the manuscript. QW reviewed and edited the manuscript. All authors read and approved the final manuscript.

## Conflict of Interest

The authors declare that the research was conducted in the absence of any commercial or financial relationships that could be construed as a potential conflict of interest.

## Publisher’s Note

All claims expressed in this article are solely those of the authors and do not necessarily represent those of their affiliated organizations, or those of the publisher, the editors and the reviewers. Any product that may be evaluated in this article, or claim that may be made by its manufacturer, is not guaranteed or endorsed by the publisher.

## Footnotes

1
http://www.uniprot.org

2
http://www.geneontology.org/

3
http://www.genome.jp/kegg/pathway.html
